# Electric field responsive nanotransducers for glioblastoma

**DOI:** 10.1186/s42234-022-00099-7

**Published:** 2022-10-19

**Authors:** Akhil Jain, Isobel Jobson, Michaela Griffin, Ruman Rahman, Stuart Smith, Frankie J. Rawson

**Affiliations:** 1 Bioelectronics Laboratory, Division of Regenerative Medicine and Cellular Therapies, School of Pharmacy, Biodiscovery Institute, University of Nottingham, Nottingham, NG7 2RD UK; 2 Children’s Brain Tumour Research Centre, School of Medicine, Biodiscovery Institute, University of Nottingham, Nottingham, NG7 2RD UK; 3 Department of Neurosurgery, Nottingham University Hospitals, Nottingham, NG7 2UH UK

**Keywords:** Tumor Treating Fields, Inorganic nanoparticles, Electric fields, Glioblastoma

## Abstract

**Background:**

Electric field therapies such as Tumor Treating Fields (TTFields) have emerged as a bioelectronic treatment for isocitrate dehydrogenase wild-type and IDH mutant grade 4 astrocytoma Glioblastoma (GBM). TTFields rely on alternating current (AC) electric fields (EF) leading to the disruption of dipole alignment and induced dielectrophoresis (DEP) during cytokinesis. Although TTFields have a favourable side effect profile, particularly compared to cytotoxic chemotherapy, survival benefits remain limited (~ 4.9 months) after an extensive treatment regime (20 hours/day for 18 months). The cost of the technology also limits its clinical adoption worldwide. Therefore, the discovery of new technology that can enhance both the therapeutic efficiency and efficacy of these TTFields will be of great benefit to cancer treatment and decrease healthcare costs worldwide.

**Methods:**

In this work, we report the role of electrically conductive gold (GNPs), dielectric silica oxide (SiO_2_), and semiconductor zinc oxide (ZnO) nanoparticles (NPs) as transducers for enhancing EF mediated anticancer effects on patient derived GBM cells. Physicochemical properties of these NPs were analyzed using spectroscopic, electron microscopy, and light-scattering techniques.

**Results:**

*In vitro* TTFields studies indicated an enhanced reduction in the metabolic activity of patient-derived Glioma INvasive marginal (GIN 28) and Glioma contrast enhanced core (GCE 28) GBM As per our journal style, article titles should not include capitalised letters unless these are proper nouns/acronyms. We have therefore used the article title “Electric field responsive nanotransducers for glioblastoma” as opposed to “Electric Field Responsive Nanotransducers for Glioblastoma” as given in the submission system. Please check if this is correct.cells in groups treated with NPs *vs.* control groups, irrespective of NPs dielectric properties. Our results indicate the inorganic NPs used in this work enhance the intracellular EF effects that could be due to the virtue of bipolar dielectrophoretic and electrophoretic effects.

**Conclusions:**

This work presents preliminary evidence which could help to improve future EF applications for bioelectronic medicine. Furthermore, the merits of spherical morphology, excellent colloidal stability, and low toxicity, make these NPs ideal for future studies for elucidating the detailed mechanism and efficacy upon their delivery in GBM preclinical models.

**Supplementary Information:**

The online version contains supplementary material available at 10.1186/s42234-022-00099-7.

## Background

Isocitrate dehydrogenase wild-type GBM is a form of highly aggressive brain tumour accounting for 49.1% of primary malignant brain tumours with less than 7% of patients surviving after 5 years post-diagnosis (Ostrom et al., 2021). The current standard of care is known as the ‘Stupp regimen’ and consists of surgical resection followed by treatment with radiotherapy and the alkylating chemotherapeutic agent, temozolomide, increasing overall survival (OS) to a median of 14.6 months (Stupp et al., 2005). Since this finding in 2005, there has been little progression in the identification of new treatments for GBM that are United States Food and Drug Administration (FDA) approved, except for TTFields. Indeed, no molecular targeted therapeutics predicated on genome biology has shown efficacy in phase III trials to date. These low intensity (<4 V/cm), intermediate frequency (100-500 kHz) and AC EFs, have been shown to further enhance cell death when in conjunction with the Stupp regimen, increasing OS by a median of 4.9 months (Stupp et al., 2017). This significant improvement to OS is not accompanied by any major side effects, with the only reported effect being contact dermatitis at the site of the electrodes.

TTFields are directional, mainly influencing cell behaviour when the electric field and axis of cell division are parallel to one another (Kirson et al., 2007). While the full extent of how TTFields work is currently unclear, two proposed mechanisms explain the mode of action: dipole alignment and DEP. In the first instance, spindle formation during mitosis is affected. Microtubules are influenced by the dipole moment of the building blocks, therefore cell division is limited (Kirson et al., 2004). In the second instance, an inhomogeneous distribution of the EF within dividing cells causes molecules to become polarised. These polar molecules then move to regions with higher EF intensities, notably the cleavage furrow in mitotic cells, and thereby interfere with cytokinesis (Kirson et al., 2007).

Despite TTFields affecting rapidly dividing cancer cells, there appears to be no effect on healthy cells with relatively slower cell division. Even cells that exhibit rapid cell division such as those found in bone marrow or intestine are not affected as they are protected by high impedance of the bone, and slower replication times compared to cancerous cells respectively (Blatt et al., 2021).

Tight junctions between epithelial cells at the blood-brain barrier (BBB) are known to inhibit the influx and efflux of many molecules to the brain (Brightman & Reese, 1969; Reese & Karnovsky, 1967). This barrier can be overcome by taking advantage of the leaky vasculature surrounding brain tumours which leads to the accumulation of NPs in the tumour, via the Enhanced Permeation and Retention (EPR) effect. NPs are frequently utilised to take advantage of this EPR effect, with studies showing that NPs of a size range of 20-100 nm are ideal to allow for maximum accumulation in the cells, tumour and longer clearance times (Perrault et al., 2009). Gold NPs (GNPs) are of particular interest for biomedical and bioelectronics applications due to their biocompatibility and tuneable properties (Shukla et al., 2005; Perrault & Chan, 2009; Sanjuan-Alberte et al., 2019). In recent years there have been numerous clinical trials utilising GNPs to treat a range of cancers, including glioblastoma (Libutti et al., 2010; Gad et al., 2012; Kumthekar et al., 2021). ZnO NPs (semiconductors) and SiO_2_ NPs (insulators), have also been well researched as a potential treatment for cancers, with the former showing the preferential killing of cancer cells over normal cells, while the latter has shown advantages of being highly tuneable, allowing for targeted drug delivery (Hanley et al., 2008; Wang et al., 2009; Murugan et al., 2017).

Electric fields have been used as external stimuli for the delivery of drugs from NPs for cancer and tissue regeneration (Kolosnjaj-Tabi et al., 2019). However, the behavior of nanoparticles under EFs in cellular environment needs further studies. Apart from the classic endocytosis mediated uptake of NPs in cancer cells, electric fields have shown to enhance the uptake of NPs by permeabilizing cancer cell membranes via electroporation and by modulating bioelectricity through voltage-gated ion channels (Aguilar et al., 2021; Chang et al., 2018). This has opened new area of research to develop new tools to study intracellular interaction of NPs with EFs. Apart from the well-known electrophoretic and dielectrophoretic movement of NPs, EFs have been shown to induce electrostatic induction and charge separation of nanomaterials. This electric polarization leads to generation of numerous bipolar nanoelectrodes which acts as transducer of EFs (Guo et al., 2021). Using modelling approaches Tiwari et al. demonstrated that spherically capped gold nanowire enhanced EFs inside the cells. They inferred that due to the uniform and homogenous distribution of EFs over nanomaterials (due to charge separation is over a short distance ≈ nm) addition source and sinks are generated. This causes a local enhancement in electric field strengths around nanomaterials in contact with cytoplasmic entities leading to the rupture of plasma membrane and eventually apoptosis (Tiwari et al., 2009).

During last decade, there has been much experiment work into the mechanism of action of TTFields, but there has been limited efforts to enhance TTFields using NPs. One example is biocompatible barium titanate NPs (BTNPs) with a high dielectric constant, which were investigated as breast cancer sensitisers in cells that were resistant to TTFields (Yoon et al., 2020). This study found that BTNPs accumulate within the cytoplasm when exposed to TTFields where they are then polarised by the inhomogeneous EF as discussed earlier, causing the BTNPs to migrate to the cleavage furrow and the cells to undergo apoptosis. While this study is the first example of using NPs to enhance TTFields, BTNPs are not FDA-approved, creating a barrier to translating these findings to a clinical setting.

Here, we investigate the underlying mechanism with the hypothesis that conductive particles would enhance the effects of TTFields. NP-enhanced TTFields mechanism of action was investigated, which is of paramount importance for the discovery of new approaches that can enhance these TTFields-induced anticancer effects. The NPs chosen were gold, zinc oxide (ZnO), and silica (SiO_2_), which are FDA-approved conductive, semi-conductive, and insulating NPs respectively (Rasmussen et al., 2010; Zhou et al., 2018). As the two suggested modes of TTFields action are due to dipole alignment and DEP, the effects of using GNPs and ZnO NPs with different electrical conductivities were chosen to address the first mechanism, while dielectric SiO_2_ NPs have potential to enhance TTFields by the second mechanism. From a clinical application perspective, the overall toxicity, pharmacokinetics, and therapeutic efficacy of the NPs must be evaluated in an accurate *in vitro* model that can reflect the cancer heterogeneity observed in clinics. Furthermore, as observed in clinics, the efficacy of TTFields varies across patients which is attributed to heterogeneity in GBM. Therefore, we utilised patient-derived GIN 28 (isolated from the invasive margin) and GCE 28 (isolated from the contrast-enhanced core), which reflect GBM tumour characteristics that are observed clinically.

## Methods

### Materials

All the reagents were of analytical grade and were used as supplied without further purification unless specified. Citrate-capped spherical GNPs, ZnO, and SiO_2_ NPs of size 50 nm were purchased from Sigma Aldrich, UK. PrestoBlue cell viability reagent and cell culture treated 22 mm coverslips (Nunc™ Thermanox™ Coverslips) were purchased from ThermoFisher Scientific, UK.

### Cell culture

GIN 28 cells were isolated from the 5-aminolevulinic acid (5ALA) fluorescing infiltrative tumour margin and GCE 28 were isolated from the core central region of a GBM patient who underwent surgery at the Queen’s Medical Centre, University of Nottingham (Nottingham, UK) using the method described earlier (Smith et al., 2017; Smith et al., 2020). Low-passage patient-derived GIN 28 and GCE 28 cells were cultured in DMEM (Gibco) supplemented with 10% FBS, 1% Penicillin/Streptomycin, and 1% L-Glutamine. Human derived cortical astrocytes (HA-COR) were obtained from ScienCell (Cat. No. 1800, Batch No. 24490) and were cultured in astrocyte medium (AM) containing 2% FBS, 1% astrocyte growth supplement, 1% Penicillin/Streptomycin from ScienCell. Cells were maintained at 37°C in an incubator with humidified atmosphere, containing 5% CO_2_. Cells were routinely tested for mycoplasma where they were grown in an antibiotic-free medium for one week before mycoplasma testing. All cells used were mycoplasma-free.

### In vitro toxicity

HA-COR, GIN 28 and GCE 28 cells were seeded in a 96-well plate at a density of 4.5 × 10^3^ cells per well and incubated for 24 hours at 37°C and 5% CO_2_. Culture media was replaced with medium containing GNPs/SiO_2_/ZnO NPs (concentration = 0.1, 0.5, 1, 2, 5, 10, 20, 50 or 100, 200 μg/ mL) and incubated for 4 hours. Next, the media was replaced with fresh media and cells were incubated for 48 or 72 hours. Finally, metabolic activity was determined using PrestoBlue^TM^. For each well, the media was replaced with media containing 10% PrestoBlue cell viability reagent and incubated at 37°C for 2 hours in an incubator before transferring the coloured metabolic product to a black-bottom 96-well plate. Finally, the fluorescence of the plate was read using a plate reader (TECAN Infinite 2000) with an excitation wavelength of 570 nm and an emission wavelength of 600 nm. Mean ± S. D values are presented relative to negative controls.

### TTFields

GIN 28 and GCE 28 cells were seeded on a 22 mm cell culture treated coverslip at a density of 3.5 × 10^4^ and incubated for 24 hours at 37°C and 5% CO_2_. Next, the media was replaced with media containing GNPs/SiO_2_/ZnO NPs at a concentration of 5 μg/mL or 25 μg/mL and incubated at 37°C for 4 hours. Next, the coverslips were transferred to ceramic Petri dishes of the inovitro™ system (Novocure, Haifa, Israel). Finally, the TTFields were applied for a duration of 48 or 72 hours using the inovitro system which consists of two pairs of perpendicular transducer arrays on the outer walls of the ceramic plate containing the Petri dishes. A sinusoidal waveform generator was attached to the transducer arrays producing alternating EFs set at a frequency of 300 kHz and 1V/cm intensity. TTFields of 300 KHz was chosen for this work, this is based on previous studies that used range of frequencies from 200-300 KHz for GBM cells (Branter et al., 1982; Linder et al., 2021). TTFields were applied bi-directionally (perpendicular to each other), which switches between the two direction every second. The temperature was measured to be 37°C inside the dishes by thermistors attached to the ceramic walls. Finally, the change in metabolic activity/ viability of GIN 28 and GCE 28 cells in response to TTFields or NP + TTFields, was determined using the PrestoBlue^TM^ assay as described above.

### Characterization

The size and morphology of the NPs were analyzed using a transmission electron microscope (JEOL 2000 FX TEM) operating at 200 kV accelerating voltage. TEM samples were prepared by dropping 15 mL of NP solution on a carbon-coated copper grid (400 Mesh, Agar Scientific), where the samples were allowed to sit on the grid for at least 15 minutes before analyses. Fourier-transform infrared spectroscopy (FTIR) was carried out by drying silica NPs at 37°C for 48 hours and finally placing the dry sample onto a Cary 630 FTIR spectrometer (Agilent Technologies Ltd) for the measurement of transmittance spectra. UV-Vis absorption spectrum of ZnO and GNPs was recorded on a Cary 3500 UV-Vis (Agilent Technologies Ltd). The hydrodynamic diameter (h_d_) and Zeta potential (ζ) of the NPs were monitored on a Malvern Zetasizer Nano-ZS (Malvern Instruments, UK).

### Statistical analysis

All the statistical analyses were performed using GraphPad Prism v9.2.0 software (GraphPad Software, Inc). All the data are expressed as mean ± S.D., unless specified. For responses that were affected by more than one variable, a two-way ANOVA with a Tukey multiple comparison post-test was used, and a p-value of ≥ 0.05 was considered significant.

## Results and discussions

### Physicochemical Properties of NPs

Inorganic NPs such as GNPs, SiO_2_, and ZnO present several advantages for biological application such as excellent biocompatibility, wide surface conjugation chemistry, and colloidal stability (Sperling et al., 2008; Hosseinpour et al., 2020; Jiang et al., 2018). Importantly due to a large difference in the dielectric constant (SiO_2_ > ZnO > GNPs) (Abdelhalim et al., 2011; Ahmad et al., 2019; Dutta & De, 2007), these NPs are best suited to gain further insight into the role of EF in GBM therapy. Previous literature indicates that nano-bio interaction depends on size; therefore, we chose spherical ~50 nm NPs to demonstrate EF effects as this NP diameter is shown to be optimum for achieving high cell uptake (Shang et al., 2014; Zhu et al., 2013; Chithrani et al., 2006; Mittag et al., 2021).

**Fig. 1:**
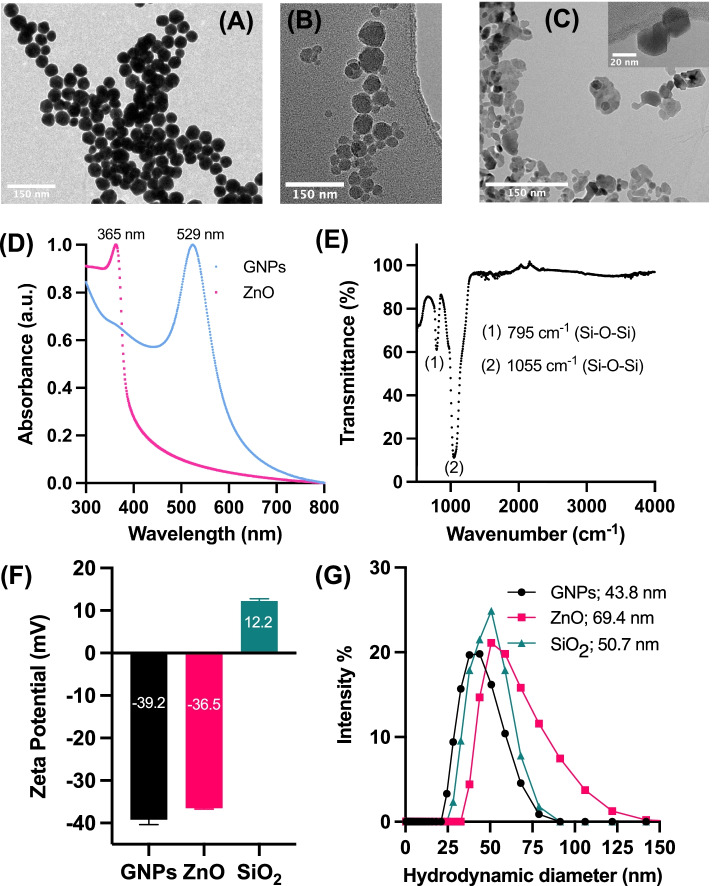
Physico-chemical characterization of inorganic NPs (Gold – GNPs; Zinc Oxide – ZnO; Silica oxide – SiO_2_). Transmission electron microscopy images of (**A**) GNPs, (**B**) SiO_2_ and (**C**) ZnO NPs. **D** UV-Vis absorption spectrum of ZnO and GNPs in PBS; **E** FTIR spectrum of SiO_2_ NPs; **F** Zeta potential; and **G** Hydrodynamic diameter obtained using DLS. Error bars represent the standard deviation of the mean n=3; N= 3

TEM analysis revealed that the mean diameter of GNPs is ~ 42 ± 4 nm (Fig. 1A) with homogenous spherical morphology, while SiO_2_ (Fig. 1B) and ZnO (Fig. 1C) were observed to agglomerate with a mean diameter of ~ 46 ± 7 nm and ~ 40 ± 11 nm, respectively. UV-Vis spectroscopy of GNPs and ZnO NPs (Fig. 1D) dispersed in phosphate buffer saline (PBS) showed distinctive absorption peaks at 529 nm and 365 nm, respectively. The absorption peak at 529 nm is attributed to surface plasmon resonance of 45 nm GNPs (Haiss et al., 2007), while the 365 nm peak in ZnO arises from the intrinsic band-gap absorption due to electron transitions from the valence band to the conduction band (O → Zn ) (Khokhra et al., 2017). Furthermore, a sharp and narrow absorption peak is a characteristic of uniform dispersion of monodisperse GNPs and ZnO NPs. FTIR of SiO_2_ NPs (Fig. 1E) showed two broad absorption peaks cantered at 795 cm^-1^ and 1055 cm^-1^ corresponding to bending vibrational modes of the Si-O-Si groups (Brassard et al., 2011). To understand the colloidal behaviour of these NPs, we carried out ζ (Fig. 1F) and dynamic light scattering (DLS) (Fig. 1G) measurements to determine their h_d_ and surface charge. Previous studies have reported that ζ and h_d_ of NPs can influence their interaction with cells. For instance, NPs with smaller h_d_ have higher diffusion constant but weak interaction with cells and vice versa with ζ (Villanueva-Flores et al., 2020). Furthermore, surface charge and h_d_ plays a key role in determining the polarization and movement of conducting and insulating NPs under the influence of EF via electrophoresis and DEP, respectively (Zhao & Bau, 2009). Therefore, it is important to balance h_d_ and ζ of the selected NPs for optimal cellular and EF interaction. In general, NPs with ζ values of ≥ - 30 mV or ≥ + 30 mV are considered to have optimal colloidal stability for biological application due to the presence of sufficient repulsive forces (Skoglund et al., 2017). Citrate capped GNPs and ZnO NPs showed a mean ζ value of – 39.2 ± 2.2 mV and -36.5 ± 1 mV which is attributed to the charge of citrate and oxide ions, respectively, suggesting good physical colloidal stability. In contrast, SiO_2_ NPs showed a positive zeta potential value of +12.2 ± 0.9 mV due to the presence of -NH_2_ groups on the surface of these NPs indicating presence of weak repulsive forces and moderate colloidal stability. DLS analysis indicated a monodisperse sample of GNPs with a h_d_ of 43.8 ± 3.2 nm. A slight increase in the size of ZnO (h_d_ = 69.4 ± 6.7 nm ) and SiO_2_ (h_d_ = 50.7 ± 5.5 nm) NPs compared to TEM measurements, further confirmed the agglomeration of these NPs in colloidal solution. Collectively, the data indicate that these inorganic NPs have optimal physicochemical properties for biological applications.

### In vitro Toxicity of NPs

**Fig. 2:**
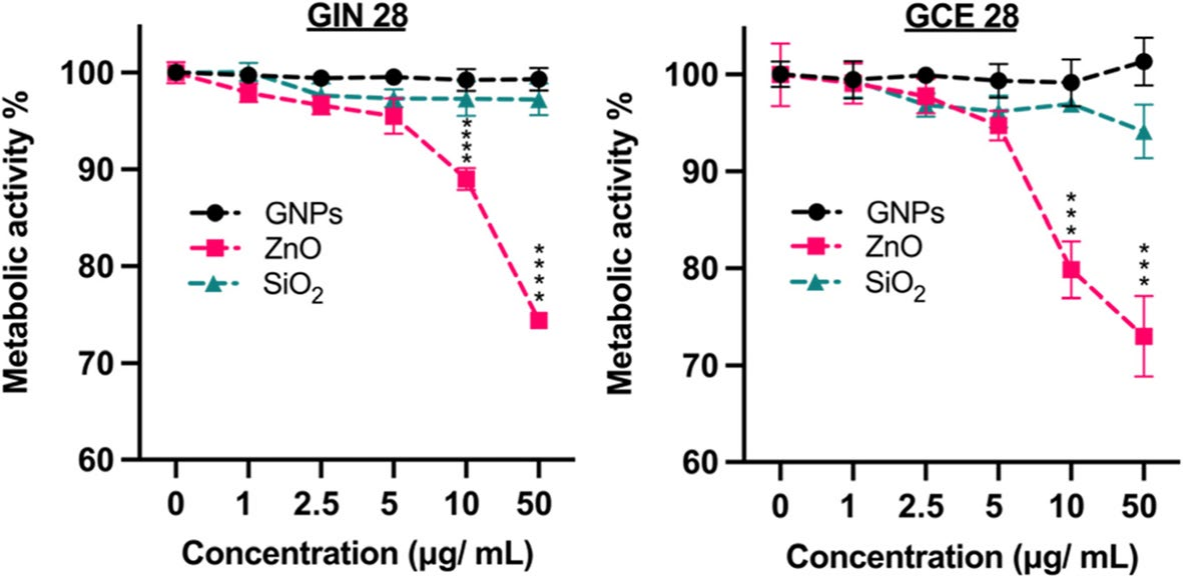
*In vitro* toxicity of inorganic NPs on patient derived GBM cells. **A** GIN 28, and **B** GCE 28 were incubated with increasing concentration of GNPs, SiO_2_ and ZnO NPs for 4 hours, before changing the media containing the NPs with fresh media. Metabolic activity was determined 48 hours after changing the media, the experiment was run in triplicate, and fluorescence at 590 nm is expressed as % of control (no NPs). Results are expressed as the mean ± S.D. *P < 0.05; **P < 0.01; ***P < 0.001; and ****P < 0.0001 obtained using 2-way ANOVA with a Tukey post-test

Before investigating the effect of using NPs in conjunction with TTFields, it was important to ascertain the toxicity of the different NPs on HA-COR (healthy cells), GIN 28 and GCE 28 cells. An experiment was therefore carried out to investigate the effect of increasing the NP concentration from 0 to 50 μg/ mL, by using PrestoBlue assay, which reports on the metabolic activity of cells as an indicator of cell viability (Peng et al., 2020; Xu et al., 2015). However, the limitation of this assay is that it does not identify the mechanism of change in metabolic activity. In the cases of GNPs and SiO_2_, there was no effect on the metabolic activity of the HA-COR (Fig. S1), GIN 28 (Fig. 2A), and GCE 28 (Fig. 2B) cells across the concentration range tested. From this, we can infer GNPs and SiO_2_ are not toxic to the healthy HA-COR and patient derived GBM cells used in this study at concentrations up to 50 μg/ mL and are therefore biocompatible. In contrast, as the concentration of ZnO NPs increased, a clear effect on cellular metabolism was observed for all cell types at higher concentrations. ZnO nanoparticles significantly reduced the metabolic activity of healthy HA-COR at a concentration of 50 μg/ mL, and at 10 μg/ mL for GIN 28 and GCE 28 cells. This decrease in metabolic activity by ZnO NPs (concentration = > 5 μg/mL) has been attributed to the generation of reactive oxygen species at higher concentrations (Liu et al., 2017). Nevertheless, the FDA has classified ZnO NPs as a “GRAS” (generally regarded as safe) at lower concentrations (Rasmussen et al., 2010). Based on the obtained data, a concentration of 5 μg/ mL of each type of NPs was chosen for the *in vitro* TTFields experiment, as at this concertation no significant change in the metabolic activity of GBM cells was observed for all three types of the NPs.

### TTFields and NPs Mediated Enhanced EF Effects in GBM Cells

**Fig. 3:**
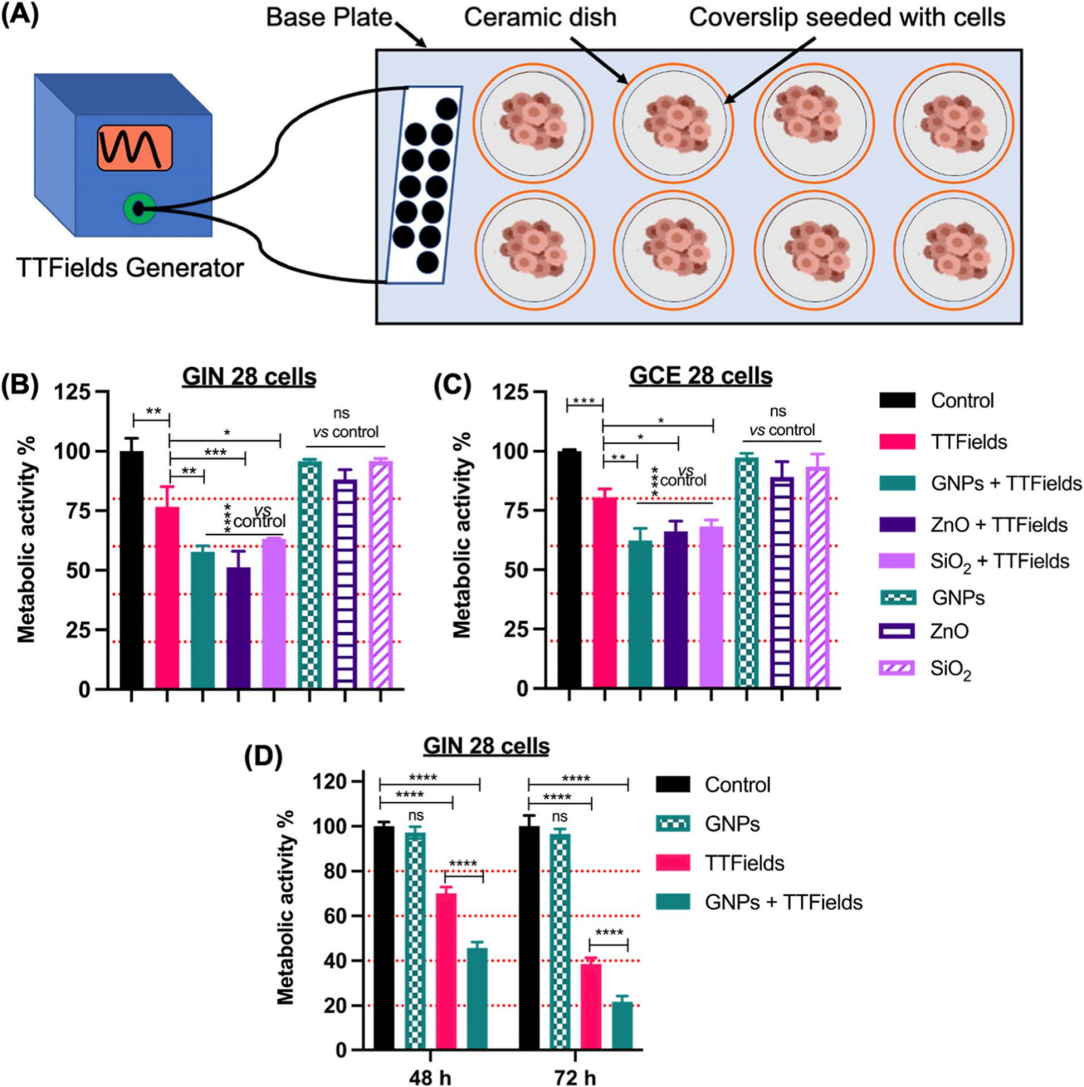
TTFields and inorganic NPs (Gold – GNPs; Zinc oxide – ZnO; Silica oxide – SiO_2_) mediated enhanced EF effects on patient-derived GBM cells. **A** Schematic representation of TTFields setup consisting of – a base plate containing 8 ceramic dishes is connected to TTFields generator via a flat cable. Cells were seeded on a coverslip placed within a ceramic dish. The base plate is placed inside an incubator where the cells were maintained at 37°C and 5% CO_2_. **B** GIN 28 cells and **C** GCE 28 cells were treated with TTFields at 300 kHz, 1V/cm, and 48 hours at NPs concentration of 5 μg/mL. **D** GNPs mediated enhanced TTFields effect on GIN 28 cells at 300 kHz, 1V/cm after 48 and 72 hours at a concentration of 25 μg/mL. Error bar represents mean ± S.E.M. from triplicate or quadruplicate repeats and two independent experiments

Encouraged by the optimal biocompatibility of ZnO NPs (≤ 5 μg/mL), GNPs (≤ 50 μg/mL) and SiO_2_ (≤ 50 μg/mL), we then investigated the role of these inorganic NPs in enhancing EF effects in patient derived GBM cells. Dielectric properties of tissues, as well as intracellular machinery, play an important role in determining the efficacy of TTFields as they are known to inhibit the proliferation of cancer cells by inducing DEP of proteins involved in the cell division process (Hershkovich et al., 2019; Wenger et al., 2015). Therefore, we hypothesised that by introducing NPs of different electrical conductivity (dielectric properties), the impedimetric properties of the cells can be modified, allowing further insights into the EF mediated cellular response. TTFields were delivered in GIN 28 and GCE 28 cells using the Inovitro laboratory system (Novocure, Haifa, Israel, schematic shown in Fig. 3A) that replicates the effect of the clinically used technique (the Optune^TM^ device). Before the application of TTFields, cells were incubated for 4 hours with either GNPs/ZnO/SiO_2_ NPs to allow uptake. We observed that the application of TTFields alone (300 kHz and1V/cm) for 48 hours decreased the metabolic activity of both GIN 28 (Fig. 3B) and GCE 28 cells (Fig. 3C) by ~ 25% *vs.* untreated control (*p* < 0.0003). Interestingly, inorganic NPs (4 hours) + TTFields (48 hours) treated showed a ~ 40% decrease in metabolic activity compared to untreated control (*p* <0.0001). Importantly, this corresponds to a ~15% higher decrease in metabolic activity compared to TTFields alone (*p* = 0.002 for GNPs; 0.0001 for ZnO, and 0.04 for SiO_2_).

Together with our previous observations and the obtained data we tentatively suggest that the inorganic NPs irrespective of their dielectric properties, could acts as EF transducers (Robinson et al., 2021). The observed enhancement with conductive GNPs and semiconducting ZnO NPs, can be further explained by the ability of these conducting and semiconducting NPs to polarize and align themselves with the applied EF to act as bipolar nanoelectrodes or transducers (Guo et al., 2021; Cao et al., 2018; Li & Anand, 2017). Furthermore, the observed enhanced electric effects could also be due to the bipolar electrophoretic effect, as both the GNPs and ZnO are negatively charged. Moreover, TTFields are known to induce biophysical forces on charged entities, this could have triggered the intracellular movement of GNPs and ZnO, thus enhancing the forces on polar intracellular structures affecting dipole alignment and disruption of mitotic spindle. In contrast, SiO_2_ is a well-known dielectric material which upon cellular uptake increases the impedance of cells. Furthermore, under the influence of TTFields (non-uniform EFs) these NPs experience dielectrophoretic forces which induces their polarization. This polarization could lead to the dielectrophoretic movement of these particles towards the pole with higher EF intensity thus enhancing the classic intracellular TTField effects (Rominiyi et al., 2021).

To further validate and establish the observed enhanced EF effect on GBM cells with GNPs regarding the conductivity of cells, we incubated GIN 28 cells with GNPs at a higher concentration (25 μg/mL) to further increase the intracellular concentration of GNPs cell conductivity. In GNP + TTFields treated group a ~52% and ~75% decrease in the metabolic activity of GIN 28 cells was observed after 48- and 72-h treatment, respectively, which was found to be significantly higher than both untreated and TTFields treated groups (Fig. 3D). Overall, the obtained data suggest that all three NPs utilized in this work enhance EF mediated anticancer effects in patient derived GBM cells. From the observed *in vitro* effects, we hypothesise that this could be due to the enhanced bipolar electrophoretic (GNPs and ZnO) and dielectrophoretic effects (SiO_2_) mediated by inorganic NPs. Further *in vitro* studies are required to elucidate the detailed molecular mechanism underlying the observed NPs mediated TTFields enhancement.

## Conclusions

In summary, conducting GNPs, semiconducting ZnO, and insulating SiO_2_ with excellent physicochemical properties, colloidal stability, and biocompatibility, were investigated as a transducer for enhancing EF activity *in vitro*. We demonstrated that inorganic NPs irrespective of dielectric permittivity, enhance the efficiency and efficacy of EFs in patient derived GBM cells isolated from intra-tumour regions. The *in vitro* efficacy was significantly enhanced by GNPs and ZnO treatment, which is attributed to the ability of these NPs to polarize and act as bipolar nanoelectrodes/ transducers which can sense external EFs, thereby enhancing electrophoretic effects. We additionally suggest that SiO_2_ NPs may enhance EF effects by increasing the forces exerted due to DEP. Furthermore, we have demonstrated that the FDA approved inorganic NPs can be used as nano transducers for enhancing intracellular EF effects. This work further paves the pathway for future studies to systemically deliver these NPs across the BBB to determine the *in vivo* efficacy.

## Supplementary Information

**Additional file 1.**

## Data Availability

Data supporting results can be found at the University of Nottingham repository (10.17639/nott.7241) and will be publicly available as of the date of publication. Any additional information required to reanalyse the data reported in this paper is available from the lead contact upon request.

## Abbreviations

EFs: Electric Fields
FTIR: Fourier-transform infrared spectroscopy
GBM: Glioblastoma
GNP: Gold nanoparticles
NPs: Nanoparticles
SiO_2_: Silicon Dioxide Nanoparticle
TEM: Transmission Electron Microscopy
TTF: Tumour treating fields
ZnO: Zinc oxide nanoparticles

## References

[CR1] Abdelhalim MAK, Mady MM, Ghannam MM (2011). Lipids Health Dis.

[CR2] Aguilar AA, Ho MC, Chang E, Carlson KW, Natarajan A, Marciano T, Bomzon ZE, Patel CB (2021). Cancers.

[CR3] Ahmad MP, Rao AV, Babu KS, Rao GN (2019). Mater Chem Phys.

[CR4] Blatt R, Davidi S, Munster M, Shteingauz A, Cahal S, Zeidan A, Marciano T, Bomzon Z, Haber A, Giladi M (2021). Front Oncol.

[CR5] Branter J, Estevez-Cebrero M, Diksin M, Griffin M, Castellanos-Uribe M, May S, Rahman R, Grundy R, Basu S, Smith S (1982). Int J Mol Sci.

[CR6] Brassard J-D, Sarkar DK, Perron J (2011). ACS Appl Mat Interfaces.

[CR7] Brightman M, Reese T (1969). J Cell Biol.

[CR8] Cao J-T, Wang Y-L, Zhang J-J, Dong Y-X, Liu F-R, Ren S-W, Liu Y-M (2018). Analytical Chem.

[CR9] Chang E, Patel CB, Pohling C, Young C, Song J, Flores TA, Zeng Y, Joubert L-M, Arami H, Natarajan A (2018). Cell Death Discov.

[CR10] Chithrani BD, Ghazani AA, Chan WC (2006). Nano Letters.

[CR11] Dutta K, De S (2007). J Nanoparticle Res.

[CR12] Gad SC, Sharp KL, Montgomery C, Payne JD, Goodrich GP (2012). Int J Toxicol.

[CR13] Guo Q, Lei C, Chen W, Zhang J, Huang B (2021). Cell Rep Phys Sci.

[CR14] Haiss W, Thanh NT, Aveyard J, Fernig DG (2007). Analytical Chem.

[CR15] Hanley C, Layne J, Punnoose A, Reddy K, Coombs I, Coombs A, Feris K, Wingett D (2008). Nanotechnology.

[CR16] Hershkovich HS, Urman N, Yesharim O, Naveh A, Bomzon ZE (2019). Phys Med Biol.

[CR17] Hosseinpour S, Walsh LJ, Xu C (2020). J Mater Chemistry B.

[CR18] Jiang J, Pi J, Cai J. Bioinorganic Chem Appl. 2018:2018. 10.1155/2018/1062562 PMC6057429 30073019

[CR19] Khokhra R, Bharti B, Lee H-N, Kumar R (2017). Sci Rep.

[CR20] Kirson ED, Dbalý V, Tovaryš F, Vymazal J, Soustiel JF, Itzhaki A, Mordechovich D, Steinberg-Shapira S, Gurvich Z, Schneiderman R (2007). Proc Nat Acad Sci.

[CR21] Kirson ED, Gurvich Z, Schneiderman Rx, Dekel E, Itzhaki A, Wasserman Y, Schatzberger R, Palti Y (2004). Cancer Res.

[CR22] Kolosnjaj-Tabi J, Gibot L, Fourquaux I, Golzio M, Rols M-P (2019). Adv Drug Delivery Rev.

[CR23] Kumthekar P, Ko CH, Paunesku T, Dixit K, Sonabend AM, Bloch O, Tate M, Schwartz M, Zuckerman L, Lezon R (2021). Science Transl Med.

[CR24] Li M, Anand RK (2017). J Am Chem Soc.

[CR25] Libutti SK, Paciotti GF, Byrnes AA, Alexander HR, Gannon WE, Walker M, Seidel GD, Yuldasheva N, Tamarkin L (2010). Clin Cancer Res.

[CR26] Linder B, Schiesl A, Voss M, Rödel F, Hehlgans S, Güllülü Ö, et al. Front Oncol. 2021:3083. 10.3389/fonc.2021.715031 PMC8361446 34395289

[CR27] Liu J, Kang Y, Yin S, Song B, Wei L, Chen L, Shao L (2017). Int J Nanomed.

[CR28] Mittag A, Hoera C, Kämpfe A, Westermann M, Kuckelkorn J, Schneider T, Glei M (2021). Toxics.

[CR29] Murugan C, Venkatesan S, Kannan S (2017). ACS Omega.

[CR30] Ostrom QT, Cioffi G, Waite K, Kruchko C, Barnholtz-Sloan JS (2021). Neuro Oncol.

[CR31] Peng H, Borg RE, Dow LP, Pruitt BL, Chen IA (2020). Proc Nat Acad Sci.

[CR32] Perrault SD, Chan WC (2009). J Am Chem Soc.

[CR33] Perrault SD, Walkey C, Jennings T, Fischer HC, Chan WC (2009). Nano Letters.

[CR34] Rasmussen JW, Martinez E, Louka P, Wingett DG (2010). Exp Opin Drug Delivery.

[CR35] Reese T, Karnovsky MJ (1967). J Cell Biol.

[CR36] Robinson AJ, Jain A, Rahman R, Abayzeed S, Hague RJ, Rawson FJ (2021). ACS Omega.

[CR37] Rominiyi O, Vanderlinden A, Clenton SJ, Bridgewater C, Al-Tamimi Y, Collis SJ (2021). Brit J Cancer.

[CR38] Sanjuan-Alberte P, Jain A, Shaw AJ, Abayzeed SA, Domínguez RF, Alea-Reyes ME, Clark M, Alexander MR, Hague RJ, Pérez-García L (2019). ACS Appl Nano Materials.

[CR39] Shang L, Nienhaus K, Nienhaus GU (2014). J Nanobiotechnology.

[CR40] Shukla R, Bansal V, Chaudhary M, Basu A, Bhonde RR, Sastry M (2005). Langmuir.

[CR41] Skoglund S, Hedberg J, Yunda E, Godymchuk A, Blomberg E, Odnevall Wallinder I (2017). PLoS One.

[CR42] Smith SJ, Diksin M, Chhaya S, Sairam S, Estevez-Cebrero MA, Rahman R (2017). Int J Mol Sci.

[CR43] Smith SJ, Rowlinson J, Estevez-Cebrero M, Onion D, Ritchie A, Clarke P, Wood K, Diksin M, Lourdusamy A, Grundy RG (2020). Neuro-Oncology Adv.

[CR44] Sperling RA, Gil PR, Zhang F, Zanella M, Parak WJ (2008). Chemical Soc Rev.

[CR45] Stupp R, Mason WP, Van Den Bent MJ, Weller M, Fisher B, Taphoorn MJ, Belanger K, Brandes AA, Marosi C, Bogdahn U (2005). New Engl J Med.

[CR46] Stupp R, Taillibert S, Kanner A, Read W, Steinberg DM, Lhermitte B, Toms S, Idbaih A, Ahluwalia MS, Fink K (2017). JAMA.

[CR47] Tiwari PK, Kang SK, Kim GJ, Choi J, Mohamed A-A, Lee JK (2009). Japanese J Appl Phys.

[CR48] Villanueva-Flores F, Castro-Lugo A, Ramírez OT, Palomares LA (2020). Nanotechnology.

[CR49] Wang H, Wingett D, Engelhard MH, Feris K, Reddy K, Turner P, Layne J, Hanley C, Bell J, Tenne D (2009). J Mater Sci Mater Med.

[CR50] Wenger C, Salvador R, Basser PJ, Miranda PC (2015). Phys Med Biol.

[CR51] Xu M, McCanna DJ, Sivak JG (2015). J Pharmacol Toxicol Methods.

[CR52] Yoon YN, Lee D-S, Park HJ, Kim J-S (2020). Sci Rep.

[CR53] Zhao H, Bau HH (2009). J Colloid Interface Sci.

[CR54] Zhou Y, Quan G, Wu Q, Zhang X, Niu B, Wu B, Huang Y, Pan X, Wu C (2018). Acta Pharmaceutica Sinica B.

[CR55] Zhu J, Liao L, Zhu L, Zhang P, Guo K, Kong J, Ji C, Liu B (2013). Talanta.

