# Supplementary material for: Electric field responsive nanotransducers for glioblastoma
Source: Bioelectron Med. 2022 Oct 19;8:17. doi: 10.1186/s42234-022-00099-7 (PMC9580136; doi:10.1186/s42234-022-00099-7)
Supplement: Supplementary file 1 — Additional file 1. [file 42234_2022_99_MOESM1_ESM.docx]

**Supplementary Information**

**Electric Field Responsive Nanotransducers for Glioblastoma**

Akhil Jain,^1,*^ Isobel Jobson,^1^ Michaela Griffin,^2^ Ruman Rahman,^2^ Stuart Smith,^2,3^ Frankie J. Rawson^1,*^

^1^Bioelectronics Laboratory, Division of Regenerative Medicine and Cellular Therapies, School of Pharmacy, Biodiscovery Institute, University of Nottingham, Nottingham, NG7 2RD, UK, ^2^Children’s Brain Tumour Research Centre, School of Medicine, Biodiscovery Institute, University of Nottingham, Nottingham, NG7 2RD, UK,

^3^Department of Neurosurgery, Nottingham University Hospitals, Nottingham, NG7 2UH, UK

*Corresponding authors: [Frankie.Rawson@nottingham.ac.uk](mailto:Frankie.Rawson@nottingham.ac.uk) and [Akhil.Jain@nottingham.ac.uk](mailto:Akhil.Jain@nottingham.ac.uk)

**Table S1.** List of inorganic nanoparticles purchased from Sigma Aldrich and their specific characteristics provided by the vendor.

| **Nanoparticle type** | **Vendor** | **Size (nm)** | **CAS No.** |
| --- | --- | --- | --- |
| Zinc oxide, dispersion (20 wt. % in water) | Sigma-Aldrich | <100 nm TEM  ≤ 40 nm APS | 721077 |
| Silica, nanoparticles dispersion in water | Sigma-Aldrich | < 50 nm DLS | 791342 |
| Gold nanoparticles  Stabilized suspension in citrate buffer | Aldrich | 50 nm | 742007 |


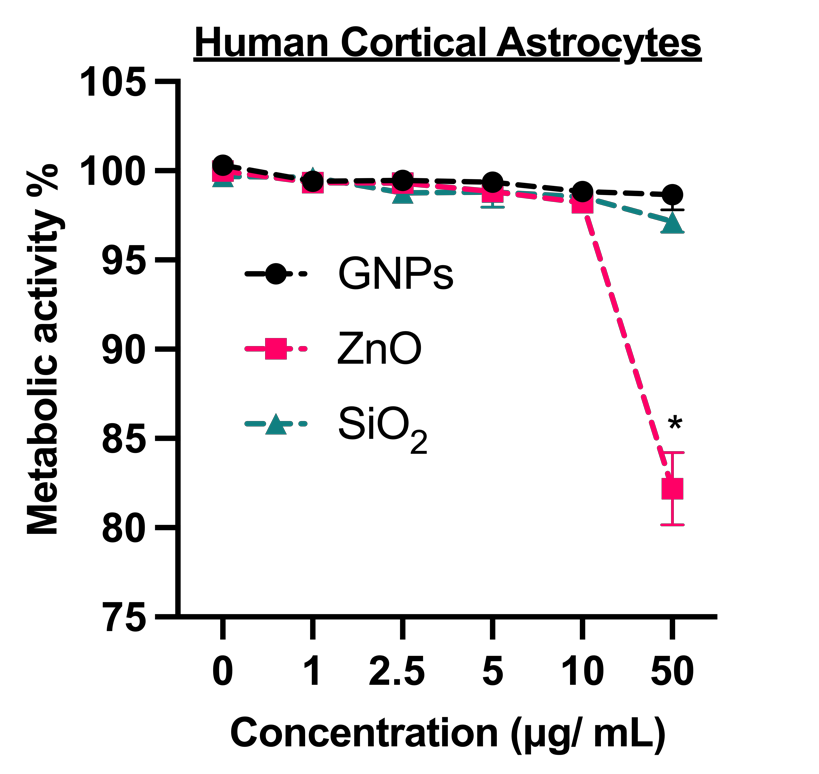


**Figure S1.** In vitro toxicity of inorganic nanoparticles on human derived cortical astrocytes (HA-COR). HA-COR were incubated with increasing concentration of GNPs, SiO_2_ and ZnO NPs for 4 hours, before changing the media containing the NPs with fresh media. Metabolic activity was determined 48 hours after changing the media using PrestoBlue assay, the experiment was run in triplicate, and fluorescence at 590 nm is expressed as % of control (no NPs). Results are expressed as the mean ± S.D. *P < 0.05 obtained using 2‐way ANOVA with a Tukey post‐test.
